# Prospective comparison among transient elastography, supersonic shear imaging, and ARFI imaging for predicting fibrosis in nonalcoholic fatty liver disease

**DOI:** 10.1371/journal.pone.0188321

**Published:** 2017-11-27

**Authors:** Myoung Seok Lee, Jeong Mo Bae, Sae Kyung Joo, Hyunsik Woo, Dong Hyeon Lee, Yong Jin Jung, Byeong Gwan Kim, Kook Lae Lee, Won Kim

**Affiliations:** 1 Department of Radiology, Seoul National University College of Medicine, Seoul Metropolitan Government Boramae Medical Center, Seoul, Korea; 2 Department of Pathology, Seoul National University College of Medicine, Seoul Metropolitan Government Boramae Medical Center, Seoul, Korea; 3 Department of Internal medicine, Seoul National University College of Medicine, Seoul Metropolitan Government Boramae Medical Center, Seoul, Korea; Medizinische Fakultat der RWTH Aachen, GERMANY

## Abstract

The diagnostic performance of supersonic shear imaging (SSI) in comparison with those of transient elastography (TE) and acoustic radiation force impulse imaging (ARFI) for staging fibrosis in nonalcoholic fatty liver disease (NAFLD) patients has not been fully assessed, especially in Asian populations with relatively lean NAFLD compared to white populations. Thus, we focused on comparing the diagnostic performances of TE, ARFI, and SSI for staging fibrosis in a head-to-head manner, and identifying the clinical, anthropometric, biochemical, and histological features which might affect liver stiffness measurement (LSM) in our prospective biopsy-proven NAFLD cohort. In this study, ninety-four patients with biopsy-proven NAFLD were included prospectively. Liver stiffness was measured using TE, SSI, and ARFI within 1 month of liver biopsy. The diagnostic performance for staging fibrosis was assessed using receiver operating characteristic (ROC) analysis. Anthropometric data were evaluated as covariates influencing LSM by regression analyses. Liver stiffness correlated with fibrosis stage (*p* < 0.05); the area under the ROC curve of TE (kPa), SSI (kPa), and ARFI (m/s) were as follows: 0.757, 0.759, and 0.657 for significant fibrosis and 0.870, 0.809, and 0.873 for advanced fibrosis. Anthropometric traits were significant confounders affecting SSI, while serum liver injury markers significantly confounded TE and ARFI. In conclusion, the LSM methods had similar diagnostic performance for staging fibrosis in patients with NAFLD. Pre-LSM anthropometric evaluation may help predict the reliability of SSI.

## Introduction

Nonalcoholic fatty liver disease (NAFLD) is the most common cause of chronic liver disease with an estimated 30% prevalence for NAFLD and 3–5% for nonalcoholic steatohepatitis (NASH) in the United States [1]. Moreover, NASH may eventually lead to hepatic complications including liver cirrhosis and hepatocellular carcinoma as well as extrahepatic comorbidities such as diabetes mellitus (DM) and cardiovascular disease. According to a recent report on the third National Health and Nutritional Examination Survey, advanced fibrosis diagnosed on the basis of noninvasive markers in NAFLD was associated with increased overall mortality, including deaths due to cardiovascular, malignancy-related, and hepatic complications [2]. Thus, the accurate assessment of liver fibrosis is critical to predict long-term outcomes and to determine treatment strategy in subjects with NAFLD [3–5].

Sonoelastography, an alternative tool for assessing liver fibrosis, is noninvasive, easily accessible, and available at point-of care, which allows the physicians to predict the risk of progression and decide treatment plans [6]. Transient elastography (TE) is the most widely used technique for evaluation of chronic liver disease and associated fibrosis. However, TE does not produce the real-time sonographic image of the liver. Moreover, TE is not feasible in some patients with morbid obesity and ascites, such as in severely obese NAFLD and decompensated NASH cirrhosis. The acoustic radiation force impulse imaging (ARFI) and the supersonic shear imaging (SSI) have several advantages over TE; they are fully integrated into a conventional ultrasound system and, thus, can be performed during routine liver sonographic examination. Both ARFI and SSI can also be used to select the examination points in the liver and evaluate heterogeneous liver fibrosis or focal liver lesions such as liver tumor [7]. Moreover, we have already evaluated the intra- and inter-observer variations of ARFI and SSI, indicating excellent agreements [8].

While the diagnostic performances of TE and ARFI for staging fibrosis in NAFLD patients have been studied [9], SSI has rarely been investigated. A previous study evaluated the diagnostic performance of SSI in comparison with those of TE and ARFI for staging fibrosis in chronic liver disease patients with heterogeneous etiologies [10], and another study compared the diagnostic performances between TE, ARFI, and SSI in white populations who had generally obese NAFLD [11]. In the most recent meta-analysis, SSI showed better diagnostic performance compared to TE for the diagnosis of severe fibrosis, although SSI did not provide significant improvements in the diagnosis of significant fibrosis and cirrhosis in NAFLD patients [12]. Regarding alcoholic liver disease patients, TE and SSI showed high diagnostic accuracies for identifying significant fibrosis and cirrhosis, with no significant difference in diagnostic performance between both elastography techniques [13]. However, to date, there has been no prospective study comparing the diagnostic performances among TE, ARFI, and SSI in Asian populations with relatively lean NAFLD.

The aims of this study were to compare the diagnostic performances of TE, ARFI, and SSI for staging fibrosis and to identify the clinical, anthropometric, biochemical, and histological features which might affect liver stiffness measurement (LSM) in a prospective NAFLD cohort.

## Materials and methods

### Study population

**Fig 1:**
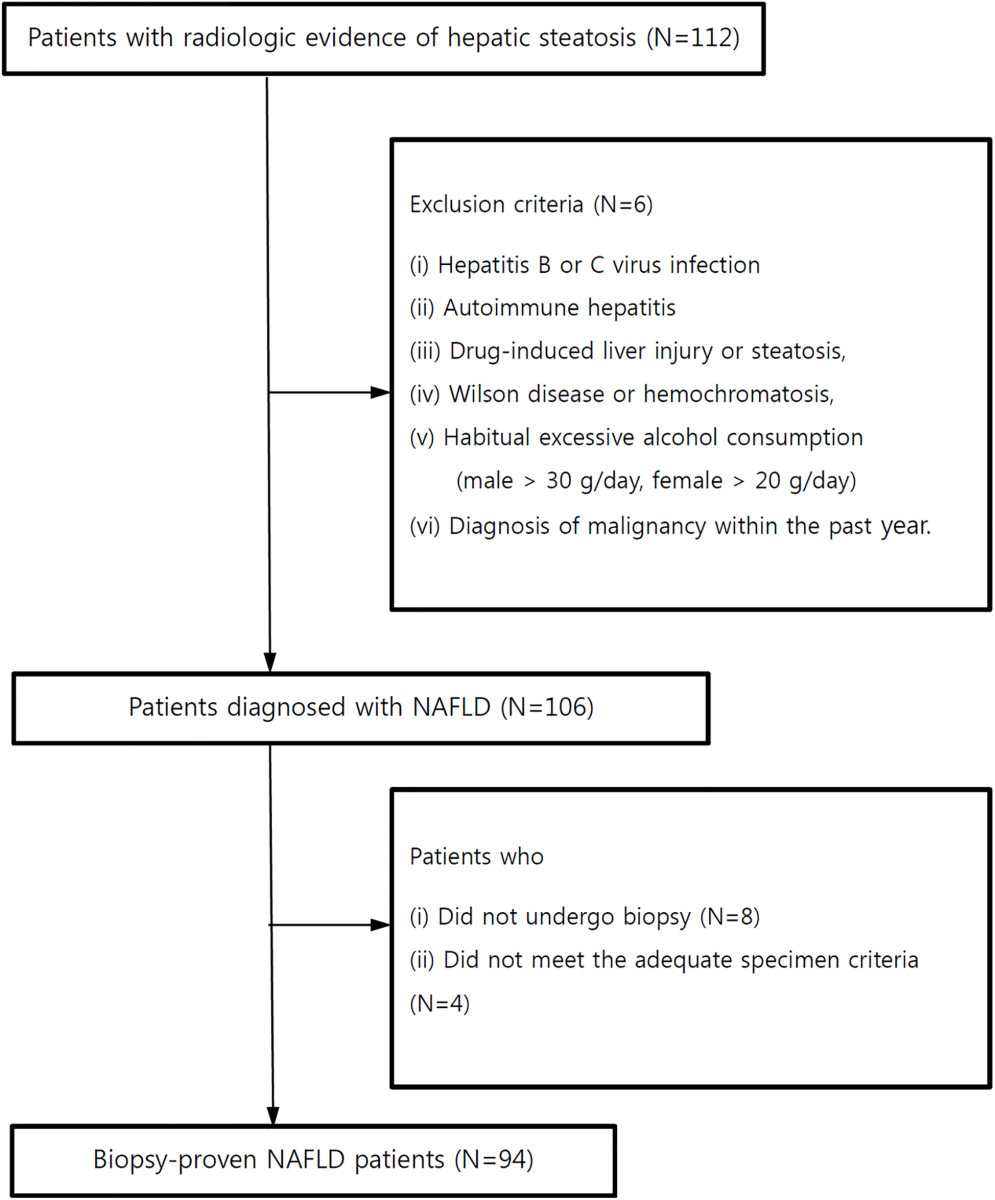
Flow diagram of the study population. Abbreviations: NAFLD, nonalcoholic fatty liver disease.

From August 2014 to November 2015, we consecutively recruited subjects with NAFLD (NCT 02206841) for this prospective study. The eligibility criteria were as follows: (i) ≥ 18 years old, (ii) bright echogenic liver on ultrasound scanning (increased liver/kidney echogenicity and posterior attenuation), and (iii) unexplained high alanine aminotransferase (ALT) levels above the reference range within the past 6 months. The exclusion criteria were as follows: (i) history of hepatitis B or C virus infection, (ii) history of autoimmune hepatitis, (iii) history of drug-induced liver injury or steatosis, (iv) Wilson disease or hemochromatosis, (v) habitual excessive alcohol consumption (male > 30 g/day, female > 20 g/day) assessed using the validated Korean version of the Alcohol Use Disorder Identification Test (AUDIT-K) questionnaire during the study period, and (vi) a diagnosis of malignancy within the past year. Of the eligible subjects, those with at least two of the following risk factors underwent liver biopsy: DM, central obesity (waist circumference ≥ 90 cm for men or ≥ 80 cm for women), a high level of triglyceride (≥ 150 mg/dL), a low level of high-density lipoprotein (HDL)-cholesterol (< 40 mg/dL for men or < 50 mg/dL for women), presence of insulin resistance, hypertension, or clinically suspected NASH or fibrosis [14]. A total of 112 subjects with radiologic evidence of hepatic steatosis were initially approached. Among them, six participants were excluded due to concurrent hepatitis B (n = 5) or a history of heavy alcohol consumption (n = 1), eight subjects did not undergo liver biopsy due to failures to fulfill the eligibility criteria for liver biopsy, and four subjects did not meet the adequate biopsy specimen criteria, yielding a drop-out rate of 16%. Thus, a total of 94 subjects with biopsy-proven NAFLD were finally included in this prospective cohort study (Fig 1). This study was carried out in accordance with the 1975 Helsinki Declaration and was approved by Seoul Metropolitan Government-Seoul National University (SMG-SNU) Boramae Medical Center Institutional Review Board (26-2015-52). Written informed consent was obtained from each study participant in the study cohort.

### Biochemical and anthropometric evaluation

The clinical, anthropometric, and biochemical data of the study population were obtained on the same day as the liver biopsy. The plasma levels of aspartate aminotransferase (AST), ALT, gamma-glutamyltransferase (GGT), total cholesterol, triglyceride, HDL-cholesterol, low-density lipoprotein (LDL)-cholesterol, free fatty acid, total bilirubin, albumin, glucose, glycosylated hemoglobin (HbA1c), insulin, c-peptide, and high sensitivity C-reactive protein (hs-CRP) were measured using 12-hour overnight fasting blood samples. Platelet count and international normalized ratio of prothrombin time (PT-INR) were tested using whole blood. Insulin resistance was determined using the homeostasis model assessment of insulin resistance (HOMA-IR). The presence of insulin resistance was defined as HOMA-IR ≥ 2 as described elsewhere [15,16]. The noninvasive serum fibrosis tests, such as AST-to-ALT ratio (AAR), AST-to-platelet ratio index (APRI), and fibrosis 4 index (FIB-4), were calculated from baseline demographic and biochemical data as described elsewhere [17–19]. Anthropometric data including total body muscle and fat mass (kg) were collected using the InBody 330 body composition analyzer (InBody, Seoul, Korea). Waist circumference (WC) was also measured. Abdominal total adipose tissue (TAT), visceral adipose tissue (VAT), and subcutaneous adipose tissue (SAT) areas were measured using non-contrast computed tomography (CT) within 1 month of percutaneous liver biopsy. The subjects were examined with a 128-detector CT scanner (Ingenuity CT, Philips Medical Systems, Cleveland, OH, USA) in the supine position. The TAT, VAT, and SAT areas were measured at the level of umbilicus with commercially available CT software (Rapidia 2.8; INFINITT, Seoul, Korea) that electronically determined the adipose tissue area by setting the attenuation values for a region of interest (ROI) within a range of −250 to −50 Hounsfield units.

### Liver stiffness measurement

Simultaneously, TE, ARFI, and SSI were conducted for LSM after at least 2-hour fast and within 1 month of percutaneous liver biopsy.

#### TE

Transient elastography (TE, Echosens, Paris, France) was performed with the Fibroscan^®^ system using the M probe. The examinations were carried out by a well-trained radiologic technician (with an experience of more than 1,000 cases of TE LSM) blinded to ARFI and SSI results and histological data. As previously described [11, 20], poorly reliable or unreliable data were defined as an interquartile range (IQR) per median of LSM (IQR/M) > 0.3 with a median LSM ≥ 7.1 kPa, and those unreliable results were excluded from analysis.

#### ARFI

The ARFI imaging was conducted by two experienced radiologists (H.W. with 13 year-experience for abdominal ultrasound; M.S.L. with 10 year-experience for abdominal ultrasound) blinded to TE and SSI results and histological data, using Acuson S2000 (Siemens AG, Erlangen, Germany). Since the inter-observer agreement of ARFI imaging proved highly reliable (0.927–0.958) in our center [15], repeat measurements between the two radiologists were not performed. The patients were required to be supine with their right arms raised overhead to increase the intercostal acoustic window. An ARFI-integrated convex probe (5C1) was positioned in the intercostal space perpendicular to the liver capsule to properly visualize the right lobe of the liver in the optimal acoustic window. The 10 × 5 mm ROI cursor was positioned in the area of liver parenchyma deeper than 2 cm from the liver capsule and free from large blood vessels, reverberation artifacts, and acoustic shadowing. Ten valid measurements at the same area were obtained from each patient during their late expiratory phases with breath-hold and the median value expressed in meters per second (m/s) was regarded as a representative value of liver stiffness. When an IQR/M was > 0.3 if the LSM was > 1.5 m/s, the measurement was considered unreliable [21].

#### SSI

The SSI (AiXplorer, Aix-en-province, France) with an SSI integrated convex probe (SC6-1) system was used for measuring shear-wave speed. One of the authors (W.K. with 12-year experience of liver ultrasound) who were not involved in performing ARFI measurement and were blinded to TE and ARFI results and histological data implemented SSI. Patient position, probe location, number of measurements, breath-holding, general rules of ROI cursor (Q-Box^™^) positioning, and the definition of unreliable measurement for SSI were similar to those for ARFI [21]. For acquiring valid measurements, the operator located a 25 × 20 mm shear-wave imaging (SWI) box at vessel- and reverberation-free liver parenchyma and waited for at least three seconds for the elastogram to be stable, and finally put the 15 mm diameter Q-Box in the area of relatively uniform elasticity, which was seen as a uniform colored area in the SWI box. For each patient, ten consecutive LSMs were obtained within one Q-Box [22].

### Liver histology

Liver biopsy as a reference standard for suspected NAFLD was performed for all study participants. Every biopsy was performed under ultrasound guidance, and in the same or similar location as the elastography measurements. The adequate liver specimen criteria were as follows: (i) ≥ 20 mm in length and (ii) ≥ eight portal tracts [23]. Liver specimens were fixed with 4% formalin and embedded in paraffin. All liver specimens stained with hematoxylin-eosin and Masson’s trichrome were analyzed by an experienced pathologist who was blinded to clinical data. Biopsy-proven NAFLD was defined as the presence of ≥ 5% steatosis [24]. Fibrosis was staged according to a 5-point scale: F0, no fibrosis; F1, perisinusoidal or portal; F2, perisinusoidal and portal/periportal; F3, septal or bridging fibrosis; and F4, cirrhosis [25]. Significant fibrosis was defined as ≥ F2 and advanced fibrosis as ≥ F3. The NAFLD activity score (NAS) ranged from 0 to 8 according to the grades of steatosis (0–3), lobular inflammation (0–3), and hepatocellular ballooning (0–2) [25] according to Brunt’s criteria [24, 26].

### Statistical analysis

Quantitative data was compared using the paired *t*-test or Wilcoxon signed rank test according to the data distribution. Proportions were compared using the chi-square test. The clinical, anthropometric, and biochemical parameters that affected the failure or unreliability of LSM were analyzed using logistic regression analysis. Spearman correlation analysis was used to evaluate the relationship between individual LSM values and the clinical, anthropometric, biochemical, and histological parameters. Successive multiple linear regression analysis was conducted to identify the independent confounders among the multiple parameters that were significantly associated with each LSM on correlation analysis (*p* < 0.05).

Receiver operating characteristic (ROC) curve analysis was performed to evaluate the diagnostic performances of TE, ARFI, and SSI for staging fibrosis. The areas under the ROC curves (AUROCs) and the 95% confidence intervals (CI) of the AUROCs were calculated for the detection of significant fibrosis, advanced fibrosis, and cirrhosis. Sensitivity, specificity, positive predictive values (PPVs), and negative predictive values (NPVs) were calculated from the AUROC curves. The optimal cut-off of each LSM method was based on the highest Youden’s index. The AUROCs for staging fibrosis were compared among TE, ARFI, and SSI using the DeLong test. These statistical analyses were performed using the IBM SPSS Statistics Ver. 20.0 (IBM Inc., Armonk, NY, USA) and MedCalc software ver. 16.2.1 (Medcalc Software BVBA, Belgium).

## Results

### Baseline clinical and histological characteristics

The mean body mass index (BMI) of 94 included patients was 27.1 kg/m^2^; 31.9% (n = 30) had normal BMI (< 25 kg/m^2^), 44.7% (n = 42) were obese (25–30 kg/m^2^), and 23.4% (n = 22) were severely obese (≥ 30 kg/m^2^). Among the study participants, 14.9% had no fibrosis, 36.2% had F1, 20.2% had F2, 12.8% had F3, and 14.9% had cirrhosis. According to Brunt’s criteria, 26.6% had severe steatosis, 41.5% had mild steatosis, and 31.9% had moderate steatosis (Table 1). In terms of the NAS, 30 out of 94 patients (31.9%) had definite NASH (NAS≥5), and 44 patients (46.8%) had borderline NASH (NAS 3 or 4). The mean values with 95% CI of individual elastographic techniques according to NAS are also presented in Table 1.

**Table 1:** Baseline characteristics of study population.

Variables	Mean (95% CI) or n (%)
**Sex**	-
**Male**	41 (43.6%)
**Female**	53 (56.4%)
**Age, year**	55.5 (52.9–58.1)
**BMI, kg/m^2^**	27.1 (26.4–27.9)
**< 25**	30 (31.9%)
**25–29.9**	41 (42.6%)
**≥ 30**	23 (25.5%)
**Waist circumference, cm**	93.4 (91.4–95.4)
**Body composition, kg**	-
**Total body fat mass**	23.8 (22.2–25.3)
**Total body muscle mass**	25.4 (24.2–26.7)
**Abdominal adipose tissue area, cm^2^**	-
**TAT**	353.6 (331.8–375.4)
**SAT**	210.9 (194.2–227.6)
**VAT**	142.6 (132.8–152.3)
**Diabetes**	37 (39.4%)
**Hypertension**	42 (44.7%)
**AST (IU/L)**	42.3 (37.9–47.6)
**ALT (IU/L)**	50.1 (42.3–57.9)
**GGT (IU/L)**	79.9 (50.4–109.3)
**Total bilirubin, mg/dL**	0.9 (0.8–1.0)
**Platelet count, x 10^9^/L**	216.8 (203.4–230.2)
**Prothrombin Time, INR**	1.07 (1.05–1.09)
**Albumin, g/dL**	4.2 (4.1–4.3)
**HbA1c, %**	6.00 (5.60–6.80)
**Insulin, μIU/mL**	14.0 (12.4–15.4)
**HOMA-IR**	4.10 (3.52–4.67)
**Fibrosis stage**	-
**F0**	14 (14.9%)
**F1**	34 (36.2%)
**F2**	19 (20.2%)
**F3**	13 (12.8%)
**F4**	14 (14.9)
**Steatosis grade**	-
**5–33%**	39 (41.5%)
**34–66%**	30 (31.9%)
**> 66%**	25 (26.6%)
**TE**	-
**Liver stiffness (kPa)**	10.6 (7.8–13.4)
**NAS: 0–2**	12.5 (0.9–24.1)
**NAS: 3–4**	8.8 (6.7–10.9)
**NAS: ≥ 5**	12.2 (6.3–18.1)
**ARFI**	-
**Liver stiffness (kPa)**	1.33 (1.24–1.43)
**NAS: 0–2**	1.23 (1.06–1.41)
**NAS: 3–4**	1.32 (1.18–1.48)
**NAS: ≥ 5**	1.41 (1.22–1.60)
**SSI**	-
**Liver stiffness (m/s);(kPa)**	2.01 (1.89–2.13); 13.9 (12.1–15.8)
**NAS: 0–2**	1.82 (1.58–2.06); 11.2 (7.8–14.6)
**NAS: 3–4**	2.02 (1.86–2.18); 13.6 (11.3–15.9)
**NAS: ≥ 5**	2.13 (1.85–2.41); 16.2 (11.9–20.6)

Abbreviations: BMI, body mass index; TAT, total adipose tissue; SAT, subcutaneous adipose tissue; VAT, visceral adipose tissue; AST, aspartate transaminase; ALT, alanine transaminase; GGT, gamma-glutamyltransferase; HOMA-IR, homeostasis model assessment of insulin resistance; TE, transient elastography; ARFI, acoustic radiation force impulse imaging; SSI; supersonic shear imaging; NAS, nonalcoholic fatty liver disease activity score.

### Parameters affecting the failure or unreliability of LSM using TE, ARFI, and SSI

The proportions of the unreliable or failed data as assessed by all the LSM methods are presented in Table 2. The unreliability or failure rate of ARFI (11.7%) was significantly lower than that of SSI (26.6%) (*p* = 0.01). However, there was no significant difference in the unreliability or failure rate of LSM between TE and ARFI or between TE and SSI. For the reliability of LSM in each stage of fibrosis, ARFI showed the significantly higher reliability rate of LSM compared to TE and SSI in NAFLD patients with no or mild fibrosis (F0–1), and compared to SSI in those with F2. Otherwise, all the LSM methods showed similar reliability in LSM rates in patients with advanced fibrosis or cirrhosis.

**Table 2:** Reliability of TE, SSI, and ARFI.

N = 94	TE	SSI	ARFI	TE vs SSI*	SSI vs ARFI*	ARFI vs TE*
**Unreliable or Failure**	20 (21.3%)	25 (26.6%)	11 (11.7%)	0.40	***0*.*01***	0.08
**Reliable**	74 (78.7%)	69 (73.4%)	83 (88.3%)	-	-	-
**Number of reliable LSMs / number of all LSMs in each fibrosis stage (%)**						
**Fibrosis stage**	-	-	-	-	-	-
**F0-1**	36/48 (75.0%)	38/48 (79.2%)	45/48 (93.8%)	0.62	***0*.*04***	***0*.*01***
**F2**	16/19 (84.2%)	11/19 (57.9%)	18/19 (94.7%)	0.08	***0*.*01***	0.30
**F3**	11/13 (84.6%)	10/13 (76.9%)	12/13 (92.3%)	0.62	0.29	0.55
**F4**	12/14 (85.7%)	10/14 (71.4%)	8/14 (57.1%)	0.37	0.43	0.10

* Presented as *p* value. Values under 0.05 were marked with bold italic.

Abbreviations: TE, transient elastography; ARFI, acoustic radiation force impulse imaging; SSI, supersonic shear imaging.

The logistic regression analysis demonstrated that the reliability of TE was affected by the diverse clinical and biochemical parameters including age, hypertension (especially, systolic blood pressure), LDL-cholesterol, liver function-related markers (AST, albumin, and platelet), and DM-related prognostic markers (insulin and HbA1c). The reliability of ARFI was influenced by age and WC. The reliability of SSI was subject to anthropometric traits such as body fat mass, BMI, TAT, SAT, and the VAT-to-SAT ratio (VSR), while it was not significantly affected by age or liver function-related serum markers (Table 3).

**Table 3:** Factors affecting the liver stiffness measurement unreliability or failure.

		Nagelkerke R^2^	Odds ratio	95% CI	*p* value
TE	Age	0.290	1.114	1.050–1.182	0.000
	HTN	0.114	3.519	1.314–9.423	0.012
	SBP	0.093	1.033	1.003–1.063	0.030
	WC	0.163	1.082	1.026–1.141	0.004
	LDL-cholesterol	0.133	0.978	0.962–0.995	0.009
	AST	0.273	1.053	1.020–1.087	0.001
	Albumin	0.285	0.012	0.001–0.132	0.000
	Platelet	0.351	0.978	0.967–0.990	0.000
	Insulin	0.203	1.160	1.057–1.273	0.002
	HbA1c	0.106	1.892	1.094–3.273	0.023
ARFI	Age	0.108	1.071	1.004–1.143	0.036
	WC	0.143	1.093	1.021–1.170	0.011
SSI	Body fat mass	0.084	1.076	1.011–1.455	0.021
	BMI	0.065	1.148	1.005–1.145	0.042
	VSR	0.101	0.126	0.020–0.773	0.025
	TAT	0.082	1.326	1.042–1.688	0.022
	SAT	0.181	1.843	1.279–2.655	0.001

Abbreviations: TE, transient elastography; ARFI, acoustic radiation force impulse imaging; SSI, supersonic shear imaging; HTN, Hypertension; SBP, systolic blood pressure; WC, waist circumference; LDL, low-density lipoprotein; AST, aspartate transaminase; BMI, body mass index; VSR, visceral-to-subcutaneous adipose tissue ratio; TAT, total adipose tissue; SAT, subcutaneous adipose tissue.

### Correlation between the two shear-wave-based elastographies

**Fig 2:**
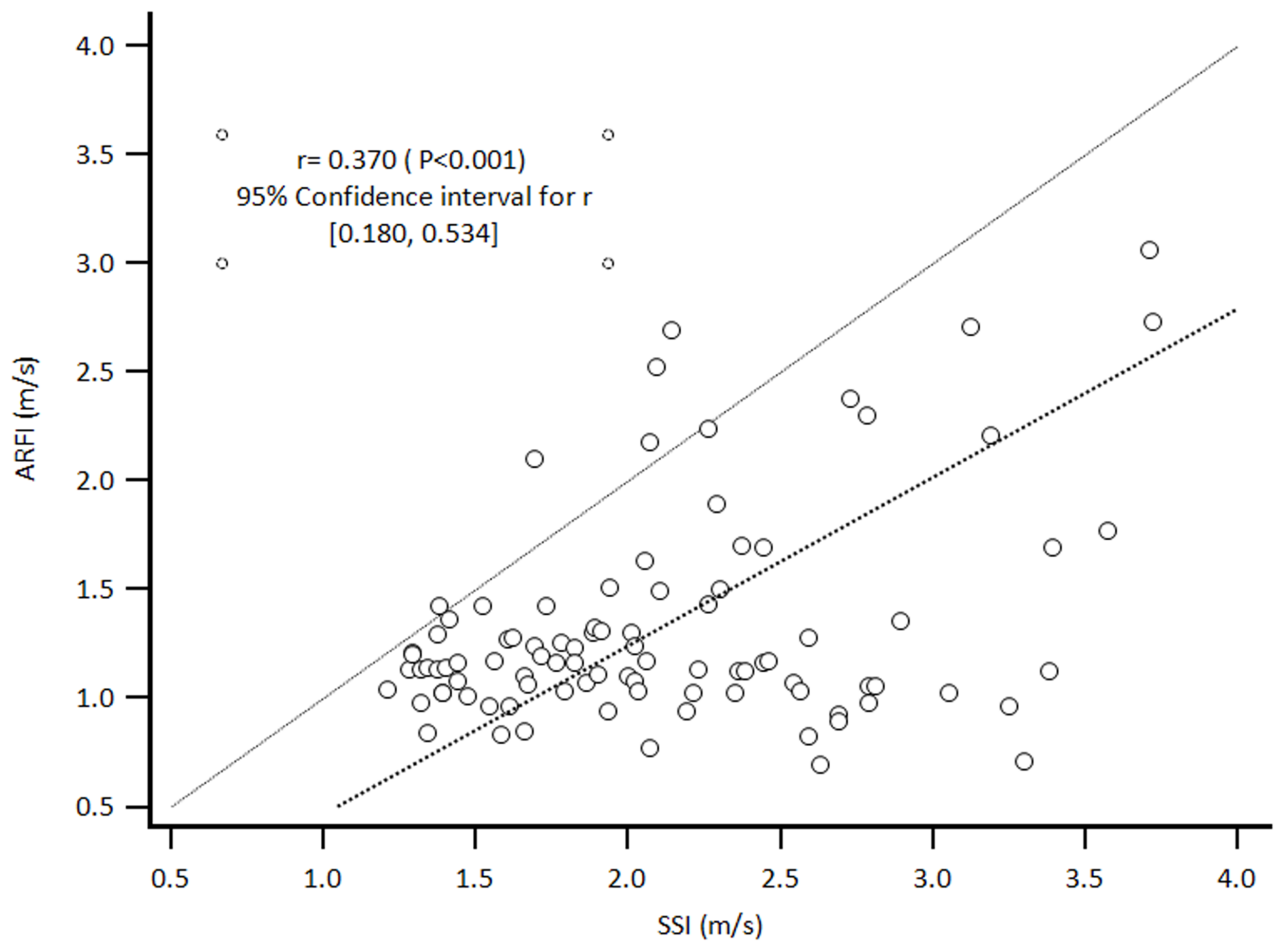
Scattered diagram showing the correlation of shear-wave velocity (m/s) between ARFI and SSI. Abbreviations: ARFI, acoustic radiation force impulse imaging; SSI, supersonic shear imaging.

There was a weak positive correlation (Spearman’s rho = 0.370, *p* < 0.001) between ARFI and SSI. A scattered diagram (Fig 2) also showed that the shear-wave velocity of SSI was generally slightly higher than that of ARFI, as mentioned in the previous study [8].

### Diagnostic performance of LSM using TE, ARFI, and SSI for staging fibrosis

The diagnostic performances presented as AUROC of TE (kPa), SSI (both m/s and kPa), and ARFI (m/s) for staging significant fibrosis, advanced fibrosis, and cirrhosis are presented in Table 4. In terms of the AUROC analysis, all the LSM methods showed good to excellent performances for staging advanced fibrosis and cirrhosis, whereas they exhibited fair performance for significant fibrosis. The optimal cut-offs according to the highest Youden’s index and 90% fixed specificity, and associated sensitivity, specificity, PPV, and NPV are presented in Table 4.

**Table 4:** Diagnostic performance of TE, SSI, and ARFI for staging liver fibrosis.

		AUROC(95% CI)	Cutoff		Sensitivity (%) (Youden / 90%Sp)	Specificity (%) (Youden)	PPV (%) (Youden / 90%Sp)	NPV (%) (Youden / 90%Sp)
			Youden	90% Sp				
TE (kPa)	F0-1 vs. F2-4	0.757 (0.645, 0.867)	7.4	7.4	62.5 / 62.5	91.7	89.3 / 89.3	68.8 / 68.8
	F0-2 vs. F3-4	0.870 (0.774, 0.965)	8.0	11.7	82.6 / 60.9	84.9	70.4 / 73.7	91.8 / 84.2
	F0-3 vs. F4	0.882 (0.737, 0.931)	10.8	11.7	91.7 / 75.0	81.2	47.8 / 47.4	98.1 / 94.7
SSI (m/s)	F0-1 vs. F2-4	0.761 (0.644, 0.856)	1.6	2.6	87.1 / 29.0	55.3	61.1 / 69.2	72.7 / 60.7
	F0-2 vs. F3-4	0.816 (0.705, 0.899)	1.9	2.7	90.0 / 35.0	63.3	50.0 / 58.3	93.9 / 77.2
	F0-3 vs. F4	0.900 (0.804, 0.959)	2.1	2.7	90.0 / 60.0	74.6	34.6 / 50.0	97.7 / 93.0
SSI (kPa)	F0-1 vs. F2-4	0.759 (0.641, 0.854)	8.3	20.5	87.0 / 29.0	55.3	64.2 / 69.2	70.0 / 64.7
	F0-2 vs. F3-4	0.809 (0.697, 0.894)	10.7	23.2	90.0 / 35.0	61.2	48.6 / 58.3	93.8 / 77.2
	F0-3 vs. F4	0.906 (0.811, 0.963)	15.1	23.2	90.0 / 60.0	78.0	40.9 / 50.0	97.9 / 93.0
ARFI (m/s)	F0-1 vs. F2-4	0.657 (0.545, 0.758)	1.35	1.29	46.2 / 48.7	93.2	85.7 / 73.1	66.1 / 64.9
	F0-2 vs. F3-4	0.873 (0.777, 0.968)	1.43	1.36	70.0 / 90.5	93.7	77.8 / 70.0	90.8 / 90.5
	F0-3 vs. F4	0.920 (0.849, 0.990)	1.50	1.50	75.0 / 75.0	90.7	46.2 / 46.2	97.1 / 97.1

Abbreviations: Sp, specificity; TE, transient elastography; ARFI, acoustic radiation force impulse imaging; SSI, supersonic shear imaging; AUROC, area under the receiver operating characteristic curve.

**Fig 3:**
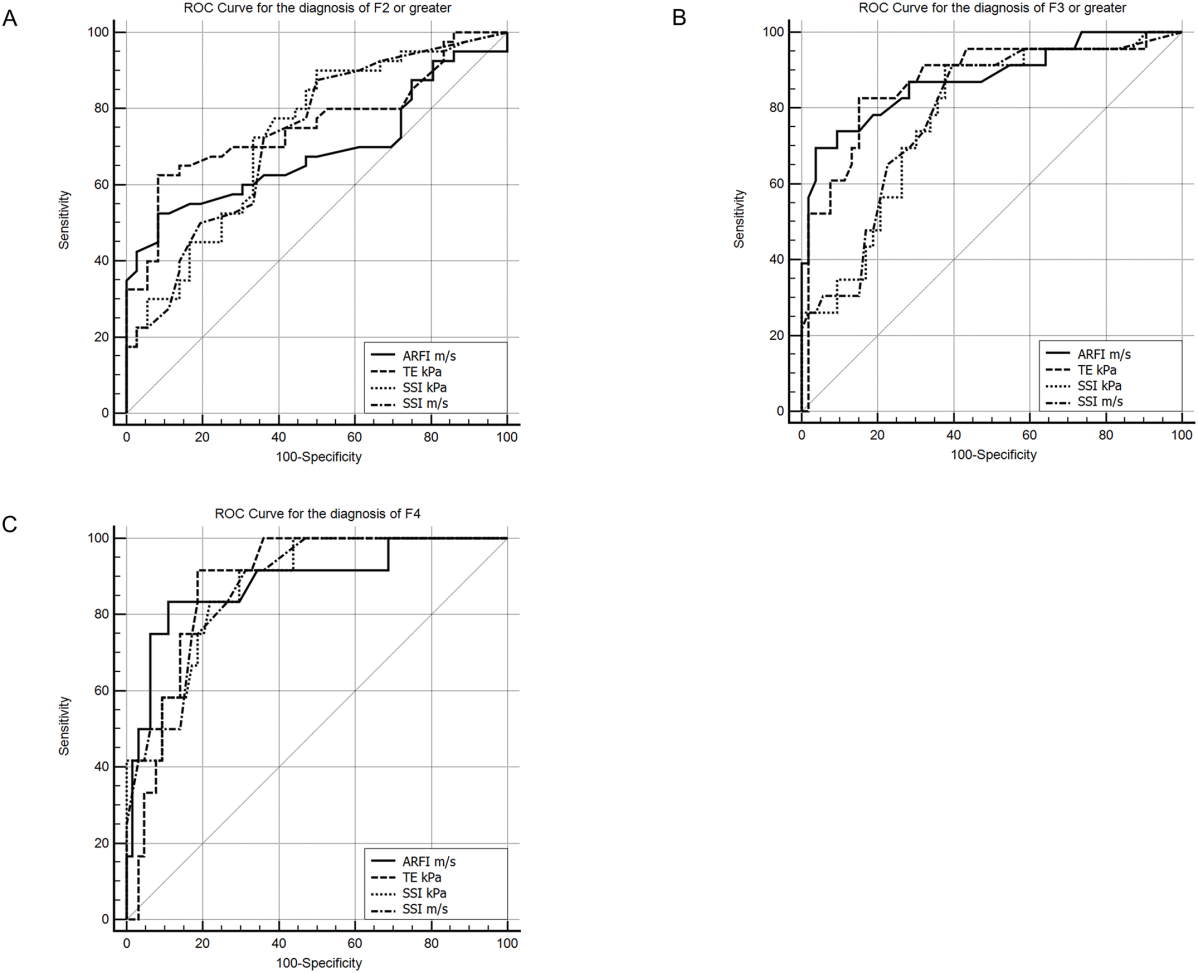
Comparative AUROCs of ARFI (m/s), TE (kPa), and SSI (kPa, m/s) for the diagnosis of each fibrosis stage. A, for significant fibrosis. B, for advanced fibrosis. C, for cirrhosis. Abbreviations: AUROC, area under the receiver operating characteristic curve; ARFI, acoustic radiation force impulse imaging; TE, transient elastography; SSI, supersonic shear imaging.

Of the individual LSM methods, SSI showed the best diagnostic performance for significant fibrosis (Table 4, Fig 3A). ARFI and TE showed better diagnostic performances than SSI for advanced fibrosis (Table 4, Fig 3B). ARFI showed the best diagnostic performance for cirrhosis (Table 4, Fig 3C). However, the diagnostic performances for staging fibrosis were not significantly different between TE, SSI, and ARFI (Table 5 and Fig 3).

**Table 5:** Pairwise comparison of diagnostic performance for staging fibrosis among TE, SSI, and ARFI.

	Fibrosis stage	*p* value			
		TE	SSI (kPa)	SSI (m/s)	ARFI
**TE**	F0-1 vs. F2-4	-	0.542	0.542	0.657
	F0-2 vs. F3-4	-	0.257	0.304	0.964
	F0-3 vs. F4	-	0.887	0.949	0.658
**SSI (kPa)**	F0-1 vs. F2-4	0.542	-	-	0.579
	F0-2 vs. F3-4	0.257	-	-	0.241
	F0-3 vs. F4	0.887	-	-	0.748
**SSI (m/s)**	F0-1 vs. F2-4	0.542	-	-	0.541
	F0-2 vs. F3-4	0.304	-	-	0.286
	F0-3 vs. F4	0.949	-	-	0.707
**ARFI**	F0-1 vs. F2-4	0.657	0.579	0.541	-
	F0-2 vs. F3-4	0.964	0.241	0.286	-
	F0-3 vs. F4	0.658	0.748	0.707	-

Abbreviations: TE, transient elastography; ARFI, acoustic radiation force impulse imaging; SSI, supersonic shear imaging.

### Comparison of LSM across fibrosis stage between TE, SSI, and ARFI

We compared LSM across the different fibrosis stages between TE, SSI, and ARFI. Liver stiffness as measured by TE, ARFI, and SSI tended to increase with fibrosis stage and showed a significantly positive correlation with fibrosis stage on Spearman correlation analysis (Table 6).

**Table 6:** Comparison of liver elasticity as measured by TE, SSI, and ARFI according to fibrosis stage.

Fibrosis stage (Number for TE/SSI/ARFI)	F0 (5/7/8)	F1 (31/31/37)	F2 (16/11/18)	F3 (11/10/12)	F4 (12/10/8)	Correlation coefficient *p* value
**TE (median, kPa)** **IQR**	4.9 (3.2, 5.5)	5.9 (4.83, 6.88)	5.85 (4.8, 7.78)	8.6 (6.7, 18.2)	15 (11.15, 20.62)	0.586 *p*<0.001
**SSI (median, m/s)** **IQR**	1.4 (1.32, 1.85)	1.6 (1.33, 2.08)	1.7 (1.55, 2.20)	2.15 (1.90, 2.20)	2.95 (2.20, 3.50)	0.532 *p*<0.001
**SSI (median, kPa)** **IQR**	5.8 (5.45, 10.63)	8.3 (5.45, 12.68)	9.6 (7.08, 14.58)	13.05 (10.4, 15.3)	29.0 (15.8, 38.3)	0.538 *p*<0.001
**ARFI (median, m/s)** **IQR**	1.12 (0.84, 1.24)	1.13 (1.02, 1.28)	1.10 (1.02, 1.35)	1.66 (1.26, 2.14)	1.98 (1.5, 2.2)	0.416 *p*<0.001

Only reliable LSM values were included in this analysis.

Abbreviations: TE, transient elastography; ARFI, acoustic radiation force impulse imaging; SSI, supersonic shear imaging; IQR, Interquartile range.

### Confounding factors influencing LSM

Fibrosis stage was significantly associated with LSM, irrespective of the LSM methods (r = 0.416–0.586, *p* < 0.001; Table 7).

**Table 7:** Factors influencing liver stiffness measurement.

	TE			SSI			ARFI		
	Rho	*p* value	R^2^	Rho	*p* value	R^2^	Rho	*p* value	R^2^
Fibrosis stage	0.586	0.000	0.167	0.532	0.000	0.180	0.416	0.000	0.459
Steatosis grade	-0.055	0.637		-0.015	0.885		-0.097	0.353	
Lobular inflammation	0.297	0.009		0.097	0.352		0.284	0.006	
Ballooning	0.207	0.073		0.131	0.207		0.172	0.097	
NAS	0.105	0.365		0.063	0.549		0.072	0.488	
NASH	0.085	0.468		0.127	0.223		0.093	0.375	
Age	0.461	0.000		0.176	0.089		0.252	0.014	
DM	0.233	0.043		0.179	0.084		0.203	0.049	
HTN	0.248	0.013		0.182	0.080		0.073	0.485	
Body muscle mass	-0.153	0.189		-0.030	0.775		-0.133	0.203	
Body fat mass	0.145	0.215		0.394	0.000		-0.145	0.166	
Weight	-0.049	0.677		0.227	0.028		-0.139	0.181	
BMI	0.167	0.150		0.393	0.000		-0.143	0.168	
WC	0.276	0.016		0.417	0.000	0.222	-0.001	0.995	
TAT	0.185	0.113		0.418	0.000		-0.148	0.157	
VAT	0.070	0.552		0.319	0.002		-0.098	0.349	
SAT	0.162	0.165		0.330	0.001		-0.142	0.176	
HDL-cholesterol	-0.114	0.326		0.152	0.145		0.019	0.854	
LDL-cholesterol	-0.280	0.014		-0.042	0.687		-0.255	0.013	
Glucose	0.236	0.040		0.199	0.054		0.176	0.090	
AST	0.375	0.001		0.184	0.075		0.364	0.000	
ALT	0.125	0.283		0.065	0.536		0.102	0.329	
Total cholesterol	-0.268	0.019		0.002	0.986		-0.280	0.006	
Triglyceride	-0.116	0.319		-0.022	0.835		-0.243	0.018	
Bilirubin	0.208	0.072		0.163	0.117		0.179	0.085	
Albumin	-0.474	0.000	0.181	-0.158	0.127		-0.217	0.035	
Platelet	-0.484	0.000		-0.222	0.068		-0.517	0.000	0.197
PT_INR	0.532	0.000		0.189	0.068		0.528	0.000	
Insulin	0.395	0.000		0.278	0.007		0.270	0.000	
GGT	0.245	0.033		0.098	0.346		0.109	0.294	
C-peptide	0.295	0.011		0.159	0.133		0.112	0.294	
HbA1c	0.252	0.028		0.148	0.153		0.135	0.193	
Free fatty acid	0.131	0.261		0.095	0.364		0.126	0.226	
hs-CRP	0.264	0.021		0.076	0.467		0.148	0.154	
HOMA-IR	0.398	0.001		0.389	0.001		0.274	0.016	
FIB-4	0.570	0.000		0.298	0.017		0.445	0.000	
APRI	0.570	0.000		0.332	0.008		0.412	0.000	
AAR	0.171	0.154		0.106	0.407		0.120	0.298	
Total R^2^	0.262			0.368			0.502		

R^2^: Multivariate model R^2^ with *p* < 0.05

Abbreviations: TE, transient elastography; ARFI, acoustic radiation force impulse imaging; SSI, supersonic shear imaging; NAS, nonalcoholic fatty liver disease activity score; NASH, nonalcoholic steatohepatitis; DM, diabetes mellitus; HTN, hypertension; BMI, body mass index; WC, waist circumference; TAT, total adipose tissue; SAT, subcutaneous adipose tissue; VAT, visceral adipose tissue; HDL, high-density lipoprotein; LDL, low-density lipoprotein; AST, aspartate transaminase; ALT, alanine transaminase; GGT, gamma-glutamyltransferase; HOMA-IR, homeostasis model assessment of insulin resistance; hs-CRP, high sensitivity C-reactive protein; FIB-4, fibrosis-4 index; APRI, AST-to-platelet ratio index; AAR, AST-to-ALT ratio.

In multivariate analysis using variables with *p* <0.05 on Spearman’s correlation analysis as independent variables, TE LSM had a possible causal relationship with fibrosis stage and serum albumin (R^2^ = 0.262, *p* < 0.001); ARFI LSM showed relationship with fibrosis stage and platelet count (R^2^ = 0.502, *p* < 0.001); SSI LSM had relationship with WC and fibrosis stage (R^2^ = 0.368, *p* < 0.001) (Table 7).

Serum fibrosis indices (HOMA-IR, FIB-4, and APRI) were significantly associated with liver stiffness as measured by all the LSM methods; however, AAR did not correlate with liver stiffness as measured by any LSM method (Table 7). The grade of steatosis had no impact on LSM, regardless of the LSM methods.

## Discussion

In this prospective biopsy-proven NAFLD cohort study, all three LSM methods (TE, SSI, and ARFI) were compared. Additionally, clinical, anthropometric, and biochemical confounders, which might affect the reliability of LSM or be correlated with LSM, were identified separately according to the different LSM methods on regression analyses.

Regarding the diagnostic performance for staging fibrosis, our results were similar to those from previous studies on TE LSM [27–29] as well as SSI and ARFI LSM [9, 11, 30]. Moreover, according to recent meta-analyses, the cut-offs for differentiating significant fibrosis, advanced fibrosis, and cirrhosis in the current study were also similar to those in the previous studies: TE (6.6~8.3 kPa for ≥ F2, 8.7~10.4 kPa for ≥ F3, and 10.3~17.5 kPa for F4) [29, 31] and ARFI (1.17~1.79 m/s for ≥ F2, 1.45~2.20 m/s for ≥ F3, and 1.61~2.90 m/s for F4) [9]. According to recent EASL-ALEH Clinical Practice Guidelines, the cut-offs of TE exam for differentiating ≥ F2 and F4 in NAFLD patients were presented as follows: 6.6~7.8 kPa for ≥ F2, and 10.3~22.3 kPa for F4 [32]. Although there was no specifically presented cut-off for NAFLD, the guidelines showed the cut-offs of ARFI for differentiating ≥ F2 and F4 in chronic liver disease patients as follows: 1.22~1.63 m/s for ≥ F2, and 1.71~4.24 m/s for F4 [32]. Aforementioned cut-offs were also similar to those in our study. For SSI, our study showed higher cut-offs than the previous studies (6.3 kPa for ≥ F2, 8.3 kPa for ≥ F3, and 10.4 kPa for F4 [11]) (7.1 kPa for ≥ F2, 9.2 kPa for ≥ F3, and 11.5 kPa for F4 [12]). It might be partially attributable to relatively low proportion of advanced fibrosis in our study compared to the previous study (27.7% vs. 43.3%) [11]. Other characteristics of our study cohort, such as pure Asian patients and relatively lower proportion of obesity, also could affect the slight increase in cut-off values during the SSI exam. However, specificities for differentiating each stage of fibrosis using SSI in the current study were not significantly different from those in the previous study (55.3%, 61.2%, and 78.0% in the current study versus 50%, 71%, and 72% in the previous study, respectively) [11].

From a clinical perspective, NAFLD patients with suspected advanced fibrosis usually require liver biopsy for accurate diagnostic and therapeutic decisions. NAFLD patients with insulin resistance and/or metabolic risk factors are at risk for advanced liver disease. Thus, sonoelastography as a tool for differentiating fibrosis stage should be sensitive for detecting advanced fibrosis to minimize undetected cases of high-risk patients who should be indicated for liver biopsy; additionally, it also should be specific for detecting significant fibrosis to obviate unnecessary biopsy and its related morbidity. Unfortunately, no LSM method sufficiently improved the diagnostic performance for detecting mild to significant fibrosis differently from advanced fibrosis and cirrhosis in the current and previous studies [9, 11, 29]. However, in the current study, TE may be occasionally preferred to ARFI or SSI under consideration of the clinical situation, such as the degree of liver injury, liver function, and anthropometric features.

The aforementioned guide for the adoption of sonoelastography was also supported by the variable reliability rates of the LSM methods according to fibrosis stage. ARFI showed a significantly higher reliability rate of LSM in patients with no to mild fibrosis (F0–1) compared to SSI and TE, and in those with F2 compared to SSI. However, the reliability rate of ARFI dropped to 57.1% in patients with cirrhosis, which was lower than the reliability rates of TE (85.7%) and SSI (71.4%), although the difference was not statistically significant. The smaller ROI box for ARFI than for TE or SSI and the heterogeneous distribution pattern of collagen deposition in the cirrhotic liver may be plausible reasons for this decline in the reliability rate of ARFI in cirrhotic patients. Regarding the shear-wave detection methods, TE generates shear wave in a direction perpendicular to skin, and detects it in the same longitudinal direction. However, in the case of both ARFI and SSI, shear wave is generated from focused ultrasound and proceeds transversely. ARFI uses multiple detection pulses perpendicular to shear wave, while SSI captures shear wave on 2D imaging using ultrafast imaging techniques. Thus, as the depth and the tissue property between skin and the target lesion may directly affect the shear wave of TE, TE seems to have lower reliability even in the mild fibrosis stage. The reason ARFI showed more reliable data in the mild fibrosis stage than SSI is unclear; however, it might be attributable to the high frame rate (350–4,000 Hz) of ultrafast imaging with SSI, which is rather insufficient to visualize the subtle change of target liver parenchyma with the slow shear wave from the mild fibrotic tissue.

The logistic regression analysis demonstrated that the reliability of TE might be easily affected by the various parameters. DM-related serum markers (Insulin and HbA1c) were significantly associated with the reliability of TE, similar to the previous study [33]. Although the previous study showed that the diagnosis with DM was associated with the failure or unreliability rate of TE, our study demonstrated that DM-related serum markers rather than DM itself were more significantly associated with the failure or unreliability rate of TE.

On the other hand, the reliability of SSI was significantly influenced by diverse anthropometric parameters. Mean BMI and SAT in the reliable SSI LSM group (26.0 kg/m^2^ and 180.94 cm^3^) were significantly lower than that in the unreliable or failure group (28.1 kg/m^2^ and 230.55 cm^3^). For TE and ARFI, WC was the only anthropometric trait that was significantly associated with the reliability of LSM. Therefore, the failure or unreliability of LSM was significantly associated with anthropometric data in all the LSM methods, as evidenced by previous studies [9–11, 29, 30, 33]. Anthropometric evaluation prior to LSM may reduce the failure or unreliability rate of all the LSM methods.

Among the confounders influencing LSM, anthropometric parameters were the most significant confounders affecting SSI LSM, while serum markers of liver injury could confound TE and ARFI LSM. However, serum insulin levels and the homeostasis model assessment of insulin resistance, which are implicated in the main pathogenesis of NAFLD, might invariably influence all the LSM methods.

In the current study, lobular inflammation on histological examination was associated with TE and ARFI LSM, while no association was observed with SSI LSM, differing from the previous study [11]. That study also showed a weak correlation of steatosis grade and NAS with TE LSM but no correlation with ARFI and SSI LSM. In the current study, steatosis severity and NAS were not significantly associated with liver stiffness as measured by any LSM method. Both TE and ARFI use the separate pulse-echo ultrasound acquisition to detect the similar shear-wave speed, while SSI uses ultrafast ultrasound imaging to detect and image shear wave. Consequently, TE and ARFI may share similar confounders affecting LSM such as liver inflammation and liver function tests.

Our study had the following inherent limitations: a relatively small sample size leading to the risk of beta error, the relative paucity of severely obese NAFLD patients in terms of BMI, and the cross-sectional study design. Only M prove was used regardless of patient habitus during the TE exam. In a recent study [34], potential misclassification of fibrosis might occur occasionally when the fasting period before performing SSI and TE was less than three hours. Thus, 2-hour fasting time also could be a substantial limitation of our study.

In conclusion, the diagnostic performances of TE, ARFI, and SSI for staging fibrosis in NAFLD patients were not statistically different in any fibrosis stage. Pre-LSM anthropometry can aid in predicting the failure or unreliability of SSI LSM.

## Supporting information

S1 Dataset All database of the included study population. (XLS)

## References

[1] Vernon G, Baranova A, Younossi ZM. Systematic review: the epidemiology and natural history of non-alcoholic fatty liver disease and non-alcoholic steatohepatitis in adults. Aliment Pharmacol. Ther. 2011;34(3):274–85. doi: 10.1111/j.1365-2036.2011.04724.x 21623852 10.1111/j.1365-2036.2011.04724.x

[2] Kim D, Kim WR, Kim HJ, Therneau TM. Association between noninvasive fibrosis markers and mortality among adults with nonalcoholic fatty liver disease in the United States. Hepatology. 2013;57(4):1357–65. doi: 10.1002/hep.26156 23175136 10.1002/hep.26156 PMC3622816

[3] Farrell GC, Larter CZ. Nonalcoholic fatty liver disease: from steatosis to cirrhosis. Hepatology. 2006;43(2 Supp 1):S99–S112 16447287 10.1002/hep.20973

[4] Angulo P, Kleiner DE, Dam-Larsen S, Adams LA, Bjornsson ES, Charatcharoenwitthaya P, et al Liver Fibrosis, but No Other Histologic Features, Is Associated With Long-term Outcomes of Patients With Nonalcoholic Fatty Liver Disease. Gastroenterology. 2015; 149(2):389–97. doi: 10.1053/j.gastro.2015.04.043 25935633 10.1053/j.gastro.2015.04.043 PMC4516664

[5] Ekstedt M, Hagström H, Nasr P, Fredrikson M, Stål P, Kechagias S, et al Fibrosis stage is the strongest predictor for disease-specific mortality in NAFLD after up to 33 years of follow-up. Hepatology. 2015;61(5):1547–54. doi: 10.1002/hep.27368 25125077 10.1002/hep.27368

[6] Lai M. Is liver stiffness measurement to stage fibrosis in patients with nonalcoholic fatty liver disease ready for clinical use? Hepatology. 2015;62(4):997–8. doi: 10.1002/hep.27902 25989922 10.1002/hep.27902

[7] Frulio N, Trillaud H. Ultrasound elastography in liver. Diagn Interv Imaging. 2013;94(5):515–34. doi: 10.1016/j.diii.2013.02.005 23623211 10.1016/j.diii.2013.02.005

[8] Woo H, Lee JY, Yoon JH, Kim W, Cho B, Choi BI. Comparison of the Reliability of Acoustic Radiation Force Impulse Imaging and Supersonic Shear Imaging in Measurement of Liver Stiffness. Radiology. 2015;277(3):881–6. doi: 10.1148/radiol.2015141975 26147680 10.1148/radiol.2015141975

[9] Liu H, Fu J, Hong R, Liu L, Li F. Acoustic Radiation Force Impulse Elastography for the Non-Invasive Evaluation of Hepatic Fibrosis in Non-Alcoholic Fatty Liver Disease Patients: A Systematic Review & Meta-Analysis. PLoS One. 2015;10:e0127782 doi: 10.1371/journal.pone.0127782 26131717 10.1371/journal.pone.0127782 PMC4489183

[10] Cassinotto C, Lapuyade B, Mouries A, Hiriart JB, Vergniol J, Gaye D, et al Non-invasive assessment of liver fibrosis with impulse elastography : Comparison of Supersonic Shear Imaging with ARFI and FibroScan. J Hepatol. 2014;61(3):550–7. doi: 10.1016/j.jhep.2014.04.044 24815876 10.1016/j.jhep.2014.04.044

[11] Cassinotto C, Boursier J, de Ledinghen V, Lebigot J, Lapuyade B, Cales P, et al Liver stiffness in nonalcoholic fatty liver disease: A comparison of Supersonic Shear Imaging, FibroScan and ARFI with liver biopsy. Hepatology. 2016;63(6):1817–27. doi: 10.1002/hep.28394 26659452 10.1002/hep.28394

[12] Herrmann E, de Lédinghen V, Cassinotto C, Chu WC, Leung VY, Ferraioli G, et al Assessment of biopsy-proven liver fibrosis by 2D-shear wave elastography: An individual patient data based meta-analysis. Hepatology. 2017 3 31 doi: 10.1002/hep.29179 [Epub ahead of print] 28370257 10.1002/hep.29179 PMC5765493

[13] Thiele M, Detlefsen S, Sevelsted Møller L, Madsen BS, Fuglsang-Hansen J, Fialla AD, et al Transient and 2-Dimensional Shear-Wave Elastography Provide Comparable Assessment of Alcoholic Liver Fibrosis and Cirrhosis. Gastroenterology. 2016;150(1):123–33. doi: 10.1053/j.gastro.2015.09.040 26435270 10.1053/j.gastro.2015.09.040

[14] Koo BK, Kim D, Joo SK, Kim JH, Chang MS, Kim BG, et al Sarcopenia is an independent risk factor for non-alcoholic steatohepatitis and significant fibrosis. J Hepatol. 2017;66(1):123–31. doi: 10.1016/j.jhep.2016.08.019 27599824 10.1016/j.jhep.2016.08.019

[15] Musso G, Gambino R, Bo S, Uberti B, Biroli G, Pagano G, et al Should nonalcoholic fatty liver disease be included in the definition of metabolic syndrome? A cross-sectional comparison with Adult Treatment Panel III criteria in nonobese nondiabetic subjects. Diabetes Care. 2008;31(3):562–8. doi: 10.2337/dc07-1526 18056890 10.2337/dc07-1526

[16] Matthews DR, Hosker JP, Rudenski AS, Naylor BA, Treacher DF, Turner RC. Homeostasis model assessment: insulin resistance and beta-cell function from fasting plasma glucose and insulin concentrations in man. Diabetologia. 1985;28(7):412–9. 3899825 10.1007/BF00280883

[17] Williams AL, Hoofnagle JH. Ratio of serum aspartate to alanine aminotransferase in chronic hepatitis. Relationship to cirrhosis. Gastroenterology. 1988;95:734–9. 3135226 10.1016/s0016-5085(88)80022-2

[18] Wai CT, Greenson JK, Fontana RJ, Kalbfleisch JD, Marrero JA, Conjeevaram HS, et al A simple noninvasive index can predict both significant fibrosis and cirrhosis in patients with chronic hepatitis C. Hepatology. 2003;38(2):518–26. doi: 10.1053/jhep.2003.50346 12883497 10.1053/jhep.2003.50346

[19] Vallet-Pichard A, Mallet V, Nalpas B, Verkarre V, Nalpas A, Dhalluin-Venier V, et al FIB-4: an inexpensive and accurate marker of fibrosis in HCV infection. comparison with liver biopsy and fibrotest. Hepatology. 2007;46(1):32–6. doi: 10.1002/hep.21669 17567829 10.1002/hep.21669

[20] Boursier J, Zarski JP, de Ledinghen V, Rousselet MC, Sturm N, Lebail B, et al Determination of reliability criteria for liver stiffness evaluation by transient elastography. Hepatology. 2013;57(3):1182–91. doi: 10.1002/hep.25993 22899556 10.1002/hep.25993

[21] Barr RG, Ferraioli G, Palmeri ML, Goodman ZD, Garcia-Tsao G, Rubin J, et al Elastography Assessment of Liver Fibrosis: Society of Radiologists in Ultrasound Consensus Conference Statement. Radiology. 2015;276(3):845–61. doi: 10.1148/radiol.2015150619 26079489 10.1148/radiol.2015150619

[22] Thiele M, Madsen BS, Procopet B, Hansen JF, Møller LM, Detlefsen S, et al Reliability Criteria for Liver Stiffness Measurements with Real-Time 2D Shear Wave Elastography in Different Clinical Scenarios of Chronic Liver Disease. Ultraschall Med. 2016 6 7. [Epub ahead of print]

[23] Schiano TD, Azeem S, Bodian CA, Bodenheimer HC Jr, Merati S, Thung SN, et al Importance of specimen size in accurate needle liver biopsy evaluation of patients with chronic hepatitis C. Clin. Gastroenterol Hepatol. 2005;3(9):930–35. 16234033 10.1016/s1542-3565(05)00541-0

[24] Brunt EM, Kleiner DE, Wilson LA, Belt P, Neuschwander-Tetri BA; NASH Clinical Research Network (CRN). Nonalcoholic fatty liver disease (NAFLD) activity score and the histopathologic diagnosis in NAFLD: distinct clinicopathologic meanings. Hepatology. 2011;53(3):810–20. 21319198 10.1002/hep.24127 PMC3079483

[25] Kleiner DE, Brunt EM, Van Natta M, Behling C, Contos MJ, Cummings OW, et al Design and validation of a histological scoring system for nonalcoholic fatty liver disease. Hepatology. 2005;41(6):1313–21. doi: 10.1002/hep.20701 15915461 10.1002/hep.20701

[26] Brunt EM, Janney CG, di Bisceglie AM, Neuschwander-Tetri BA, Bacon BR, et al Nonalcoholic steatohepatitis: a proposal for grading and staging the histological lesions. Am J Gastroenterol. 1999;94(9):2467–74. doi: 10.1111/j.1572-0241.1999.01377.x 10484010 10.1111/j.1572-0241.1999.01377.x

[27] Yoneda M, Yoneda M, Fujita K, Inamori M, Tamano M, Hiriishi H, et al Transient elastography in patients with non‐alcoholic fatty liver disease (NAFLD). Gut. 2007;56(9):1330–31. doi: 10.1136/gut.2007.126417 17470477 10.1136/gut.2007.126417 PMC1954961

[28] Wong VW, Vergniol J, Wong GL, Foucher J, Chan HL, Le Bail B, et al Diagnosis of fibrosis and cirrhosis using liver stiffness measurement in nonalcoholic fatty liver disease. Hepatology. 2010;51(2):454–62. doi: 10.1002/hep.23312 20101745 10.1002/hep.23312

[29] Abenavoli L, Beaugrand M. Transient elastography in non-alcoholic fatty liver disease. Ann Hepatol. 2012;11(2):172–8. 22345333

[30] Cassinotto C, Lapuyade B, Aït-Ali A, Vergniol J, Gaye D, Foucher J, et al Liver Fibrosis: Noninvasive Assessment with Acoustic Radiation Force Impulse Elastography—Comparison with FibroScan M and XL Probes and FibroTest in Patients with Chronic Liver Disease. Radiology. 2013;269(1):283–92. doi: 10.1148/radiol.13122208 23630312 10.1148/radiol.13122208

[31] Kwok R, Tse YK, Wong GL, Ha Y, Lee AU, Ngu MC, et al Systematic review with meta-analysis: non-invasive assessment of non-alcoholic fatty liver disease—the role of transient elastography and plasma cytokeratin-18 fragments. Aliment Pharmacol Ther. 2014;39(3):254–69. doi: 10.1111/apt.12569 24308774 10.1111/apt.12569

[32] European Association for Study of Liver, Asociacion Latinoamericana para el Estudio del Higado. EASL-ALEH Clinical Practice Guidelines: Non-invasive tests for evaluation of liver disease severity and prognosis. J Hepatol. 2015;63(1):237–64 doi: 10.1016/j.jhep.2015.04.006 25911335 10.1016/j.jhep.2015.04.006

[33] Wong GL, Wong VW, Chim AM, Yiu KK, Chu SH, Li MK, et al Factors associated with unreliable liver stiffness measurement and its failure with transient elastography in the Chinese population. J Gastroenterol Hepatol. 2011;26(2):300–5. doi: 10.1111/j.1440-1746.2010.06510.x 21261720 10.1111/j.1440-1746.2010.06510.x

[34] Kjaergaard M, Thiele M, Jansen C, Staehr Madsen B, Gortzen J, Strassburg C, Trebicka J, et al High risk of misinterpreting liver and spleen stiffness using 2D shear-wave and transient elastography after a moderate or high calorie meal. PLoS One. 2017;12:e0173992 doi: 10.1371/journal.pone.0173992 28376114 10.1371/journal.pone.0173992 PMC5380309

