# Correction: Prospective comparison among transient elastography, supersonic shear imaging, and ARFI imaging for predicting fibrosis in nonalcoholic fatty liver disease

**DOI:** 10.1371/journal.pone.0200055

**Published:** 2018-06-26

**Authors:** Myoung Seok Lee, Jeong Mo Bae, Sae Kyung Joo, Hyunsik Woo, Dong Hyeon Lee, Yong Jin Jung, Byeong Gwan Kim, Kook Lae Lee, Won Kim

The Institutional Review Board number appears incorrectly in the article. The correct Institutional Review Board number is 26-2016-40.

The penultimate sentence of the Study population sub-section in the Materials and methods section should read: This study was carried out in accordance with the 1975 Helsinki Declaration and was approved by Seoul Metropolitan Government-Seoul National University (SMG-SNU) Boramae Medical Center Institutional Review Board (26-2016-40).
